# Cisterna chyli as an optimal marker of tolvaptan response in severe cirrhotic ascites

**DOI:** 10.1038/s41598-022-11889-z

**Published:** 2022-05-17

**Authors:** Masashi Hirooka, Yohei Koizumi, Ryo Yano, Yoshiko Nakamura, Koutarou Sunago, Atsushi Yukimoto, Takao Watanabe, Osamu Yoshida, Yoshio Tokumoto, Masanori Abe, Yoichi Hiasa

**Affiliations:** Department of Gastroenterology and Metabiology, Ehime University Graduate School of Medicine, Shitsukawa 454, Toon, Ehime 791-0295 Japan

**Keywords:** Hepatology, Diagnostic markers

## Abstract

For patients with cirrhosis, no definitive predictor of the efficacy and prognosis of tolvaptan treatment exists. We assessed the cisterna chyli’s utility as an optimal marker. We retrospectively enrolled 172 patients with cirrhosis. The effect of tolvaptan was evaluated using post-treatment survival time. The overall response to tolvaptan was 52.3%. The median cisterna chyli diameter was 4.1 mm. Of 172 patients, 100 were included in the pilot set and 72 in the validation set. According to the Youden index, the cisterna chyli diameter’s cutoff value was 4 mm, with a sensitivity, specificity, positive predictive value, negative predictive value, positive likelihood ratio, and negative likelihood ratio of 92%, 83%, 86%, 91%, 5.43, and 0.09, respectively, in the pilot set. The area under the curve of the cisterna chyli diameter for evaluating tolvaptan’s effect was 0.911 and 0.988 in the pilot and validation sets, respectively. During multivariate analysis, cisterna chyli narrowing and furosemide treatment were significant predictive factors for tolvaptan’s insufficient effect. Cumulative liver transplantation-free survival rates were significantly higher in patients with cisterna chyli dilatation than in those without (p = 0.028). Our findings suggest a strong association of cisterna chyli with tolvaptan treatment response in patients with cirrhosis and hepatic edema.

## Introduction

Ascites and hepatic edema are major complications observed in patients with decompensated cirrhosis and are associated with increased morbidity^1,2^ and poor quality of life^3^. The European Association for the Study of the Liver and the American Association for the Study of Liver Diseases have provided practice guidelines for the management of ascites^3,4^. Conventional treatment predominantly includes a salt-restricted diet in conjunction with diuretics administration, such as loop diuretics and anti-aldosterone drugs^5–8^. Although these diuretics have commonly been used in patients with cirrhosis, severe ascites develops in approximately 5–10% of patients with ascites^9–11^.

Patients with cirrhosis commonly have high levels of vasopressin^12^. Vasopressin is an antidiuretic hormone that increases the reabsorption of water by increasing water permeability through the V2 receptors in the renal collecting duct^13–15^. Recently, tolvaptan has been used for severe ascites, which does not respond to conventional treatment. This selective vasopressin V2-receptor antagonist reduces vasopressin-induced water reabsorption by downregulating aquaporin-2 expression in the collecting duct without affecting electrolyte excretion^16,17^. The promising role of tolvaptan as an add-on treatment in patients with severe ascites resistant to conventional treatment has recently been proposed, as it is able to decrease body weight, alleviate edema, and promote long-term outcomes^18^. However, not all patients respond to tolvaptan. Up to 30% fail to achieve a satisfactory response to tolvaptan administration in terms of increased urination or body-weight reduction^13^. Although tolvaptan’s effect varies across patients with cirrhosis, the reason remains unclear. Certain markers have been reported to predict the effect of tolvaptan; however, the results remain inconclusive^19^.

We focused on lymphatic dysfunction as a new marker. The relationship between ascites formation and lymphatic dysfunction has recently attracted attention^20–22^. In normal-lymphatic-drainage hypofunction, interstitial fluid accumulation potentially contributes to clinical manifestations, such as lymphedema and ascites^22^. In patients with cirrhosis in the compensated phase, the lymphatic system facilitates the prevention of ascites by reabsorbing excess fluid in the hepatic, splanchnic, and intestinal areas. As a result, lymph flow is enhanced, thus promoting hepatic lymphangiogenesis^23,24^. However, in patients with advanced cirrhosis, this compensatory mechanism is not adequate to prevent the development of ascites. Lymphatic fluid originating from the space of Disse predominantly flows through the space of Mall and subsequently into lymphatic capillaries. Some of the lymphatic fluid in the space of Disse flows into the interstitium surrounding the central vein or underneath the hepatic capsule^25^. Lymphatic capillaries in the portal tract coalesce into collecting vessels and subsequently drain into the cisterna chyli and enlarged origin of the thoracic duct. Lymphatic fluid, through the thoracic duct, drains into the left subclavicular vein and returns to the systemic blood circulation^22^. During excessive lymphatic fluid drainage, lymphatic drainage dysfunction is considered to occur at one of the sites in patients with decompensated cirrhosis. Between these sites, it is feasible to detect cisterna chyli using multi-detector row computed tomography (CT)^26^.

Therefore, this study aimed to determine the utility of the cisterna chyli as an optimal marker of tolvaptan-administration response in patients with severe cirrhotic ascites.

## Results

### Baseline characteristics of patients

**Figure 1:**
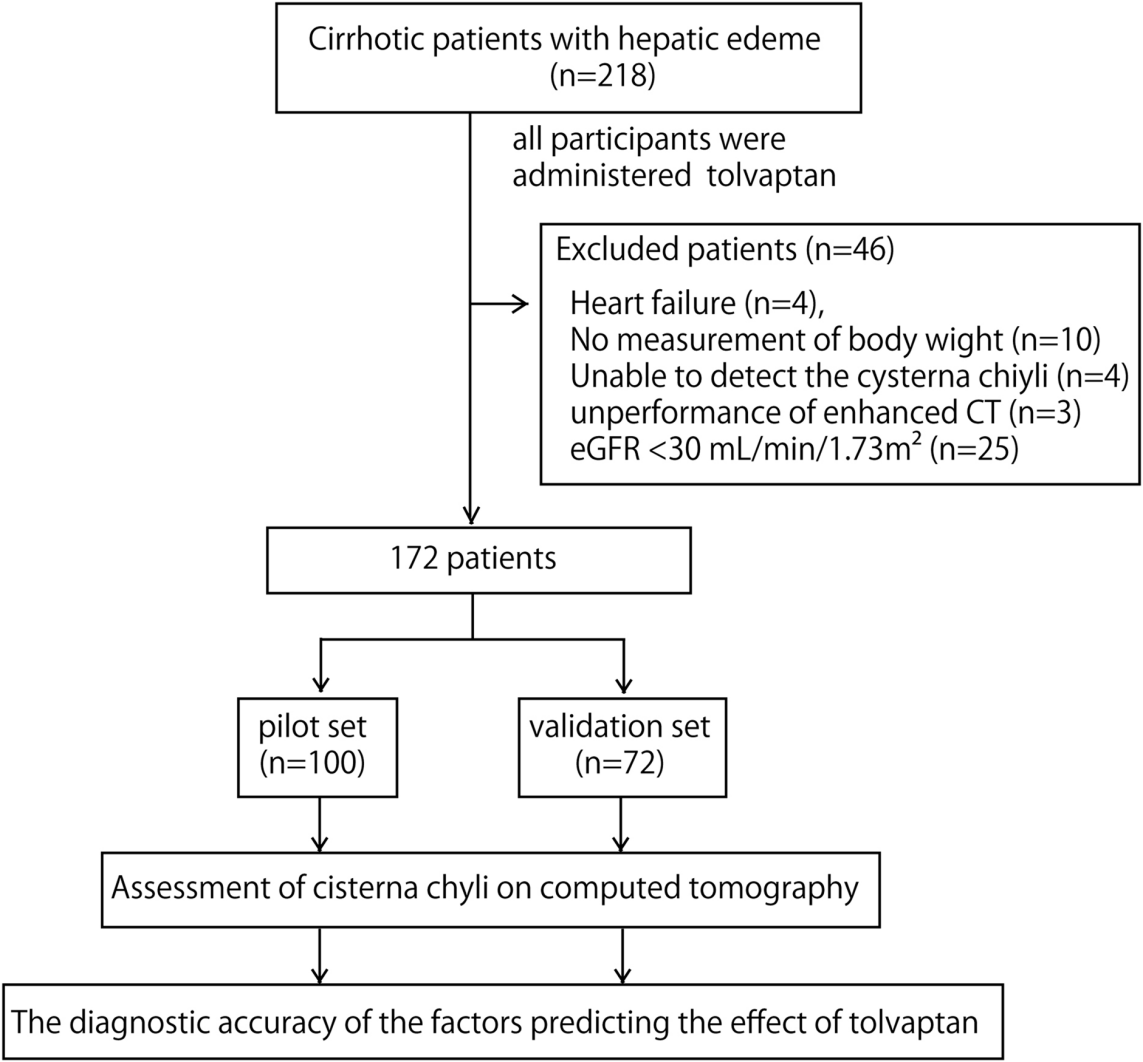
Study design.

Initially, the study included 218 patients. Patients with heart failure (n = 4), without body weight records after tolvaptan treatment (n = 10), in whom cisterna chyli could not be detected on CT (n = 4), on whom contrast-enhanced CT was not performed before tolvaptan initiation (n = 3), and with an estimated Glomerular Filtration Rate (eGFR) < 30 mL/min/1.73m^2^ (n = 25) were excluded. Finally, 172 patients were included in this study (58 women; median age, 67.0 years; Fig. 1). To define the cut-off value, 100 patients were assigned to the pilot set in the order of entry. The remaining 72 patients were assigned to the validation set to confirm the efficacy of tolvaptan. Patient demographics and clinical characteristics are shown in Table 1. The number of patients who responded to tolvaptan was 9 (11.0%) and 85 (94.4%) for patients with cisterna chyli diameters < 4 mm and ≥ 4 mm, respectively. Patient demographics and baseline characteristics between tolvaptan-responders vs. non-responders. are shown in Table 2.

**Table 1:** Patient characteristics.

Factors	All (n = 172)	Group		p value
		Pilot set (n = 100)	Validation set (n = 72)	
Median diameter of Cisterna chyli (mm)	4 (2–7)	4 (2–7)	4 (2–6)	0.710
Median age (years)	67 (58–74)	66 (56–75)	67 (60–72)	0.742
Difference in body weight (kg) from baseline	− 1.8 (− 4.2–0.1)	− 1.8 (− 4.0 to − 0.1)	− 1.9 (− 4.6–0.2)	0.684
Decrease of 1.5 kg or greater from baseline in body weight	90 (52.3%)	53 (53.0%)	37 (51.4%)	0.835
Anti-HCV ( +)	63 (36.6%)	36 (36.0%)	27 (37.5%)	0.840
Male	114 (66.2%)	67 (67.0%)	47 (65.3%)	0.814
Albumin (g/dL)	2.7 (2.3–3.0)	2.7 (2.5–3.0)	2.7 (2.2–3.0)	0.511
Total bilirubin (mg/dL)	1.9 (1.0–3.6)	1.9 (0.9–3.3)	1.9 (1.1–3.8)	0.525
ALT (U/L)	29 (20–49)	28 (20–44)	31 (21–56)	0.189
Sodium (mEq/L)	136 (132–139)	136 (132–139)	136 (132–139)	0.908
Potassium (mEq/L)	4.1 (3.7–4.5)	4.1 (3.7–4.8)	4.0 (3.7–4.5)	0.748
BUN (mg/dL)	18 (13–26)	18 (13–28)	19 (13–25)	0.918
BUN/Creatinine	21.2 (16.4–27.1)	21.2 (16.2–27.1)	21.2 (16.7–26.8)	0.858
eGFR (%)	62.8 (46.1–82.0)	62.1 (47.9–78.6)	67.8 (43.4–86.2)	0.729
CRP (mg/dL)	1.19 (0.37–3.29)	1.22 (0.42–3.53)	1.09 (0.33–2.87)	0.572
Platelet counts (10^3^/μL)	110 (69–159)	107 (67–159)	118 (73–169)	0.680
Prothrombin time (%)	58.8 (45.5–71.9)	60.8 (46.3–74.1)	56.3 (43.3–71.7)	0.371
Ammonia (μg/dL)	57 (38–84)	57 (40–83)	56 (33–88)	0.544
MELD	10.7 (5.9–14.3)	11.6 (6.3–14.2)	10.8 (5.6–14.8)	0.558
Change in urinary volume (mL) ^a^	686 (203–1156)	684 (37–1164)	700 (339–1012)	0.552
FENa	0.50 (0.24–1.04)	0.74 (0.30–1.18)	0.42 (0.21–0.88)	0.052
Urinary sodium (mmol/L)	51 (28–77)	49 (29–79)	52 (27–74)	0.430
Dose of fulosemide (mg) ^b^	20 (20–40)	20 (20–40)	20 (20–40)	0.969
Dose of spironolactone (mg) ^c^	50 (25–50)	50 (25–50)	50 (25–50)	0.492
Furosemide > 40 mg ^b^	63 (36.6%)	37 (37.0%)	26 (36.1%)	0.905
Spironolactone > 50 mg ^c^	110 (64.0%)	68 (68.0%)	42 (58.3%)	0.193
Esophagogastric varices	110 (64.0%)	58 (58.0%)	52 (72.2%)	0.076
History of HCC treatment	14 (8.1%)	8 (8.0%)	6 (8.3%)	0.937
History of liver transplantation	18 (10.5%)	13 (13.0%)	5 (6.9%)	0.201
Duration of tolvaptan treatment (days)	82 (34–172)	75 (32–190)	102 (34–156)	0.830

*HCV* hepatitis C virus, *ALT* alanine aminotransferase, *BUN* blood urea nitrogen, *eGFR* estimated glomerular filtration rate, *CRP* c-reactive protein, *MELD* model for end-stage liver disease, *FENa* fractional excretion of sodium: (urine sodium/serum sodium)/(urine creatinine/serum creatinine), *HCC* hepatocellular carcinoma.

^a^Difference in 24-h urine volume before and after tolvaptan treatment.

^b^Dose of furosemide during tolvaptan treatment.

^c^Dose of spironolactone during tolvaptan treatment.

**Table 2:** Patient characteristics for tolvaptan responders vs. non-responders.

Factors	Group		p value
	Responders (n = 90)	Non-responders (n = 82)	
Median diameter of Cisterna chyli (mm)*	6 (5–8)	2 (1–2)	< 0.001
Number of cisterna chyli ≥ 4 mm	83 (92.2%)	9 (10.1%)	< 0.001
Median age (years)	66 (56–73)	67 (58–74)	0.983
Anti-HCV ( +)	28 (31.1%)	35 (42.7%)	0.116
Male	63 (70.0%)	51 (62.2%)	0.279
Albumin (g/dL)	2.7 (2.3–2.9)	2.8 (2.5–3.1)	0.070
Total bilirubin (mg/dL)	1.7 (0.9–3.3)	2.2 (1.2–3.6)	0.092
ALT (U/L)	30 (22–56)	28 (19–44)	0.162
Sodium (mEq/L)	135 (132–139)	137 (132–138)	0.577
Potassium (mEq/L)	4.0 (3.7–4.4)	4.1 (3.7–4.5)	0.696
BUN (mg/dL)*	21 (15–29)	17 (12–23)	0.033
BUN/Creatinine*	22.5 (18.5–29.3)	19.9 (15.7–25.1)	0.025
eGFR (%)	62.0 (48.0–79.6)	64.2 (45.8–82.3)	0.824
CRP (mg/dL)	1.57 (0.36–4.33)	0.95 (0.39–2.42)	0.088
Platelet counts (10^3^/μL)	114 (77–167)	108 (63–154)	0.175
Prothrombin time (%)	64.0 (51.0–75.5)	64.3 (52.7–70.7)	0.371
Ammonia (μg/dL)	60 (38–83)	56 (37–87)	0.825
MELD	11.5 (7.2–15.0)	10.3 (5.6–14.8)	0.087
FENa	0.51 (0.23–1.17)	0.46 (0.28–1.03)	0.859
Urinary sodium (mmol/L)	48 (25–74)	54 (33–80)	0.430
Dose of fulosemide (mg)*^a^	20 (20–40)	20 (20–40)	0.049
Dose of spironolactone (mg)^b^	50 (50–50)	50 (25–50)	0.053
Furosemide > 40 mg*^a^	41 (45.6%)	22 (26.8%)	0.011
Spironolactone > 50mg^b^	56 (62.2%)	54 (65.9%)	0.620
Esophagogastric varices	61 (67.8%)	49 (59.8%)	0.076
History of HCC treatment	8 (8%)	6 (8.3%)	0.350
Chylous ascites	0	1 (1.2%)	

*HCV* hepatitis C virus, *ALT* alanine aminotransferase, *BUN* blood urea nitrogen, *eGFR* estimated glomerular filtration rate, *CRP* c-reactive protein, *MELD* model for end-stage liver disease, *FENa* fractional excretion of sodium: (urine sodium/serum sodium)/(urine creatinine/serum creatinine, *HCC* hepatocellular carcinoma.

^a^Dose of furosemide during tolvaptan treatment.

^b^Dose of spironolactone during tolvaptan treatment.

*p < 0.05.

### Diagnostic accuracy of the effect of tolvaptan

The diagnostic accuracy of the factors predicting the effect of tolvaptan in the pilot set is shown in Table 3. According to the Youden index, the cutoff value for the cisterna chyli diameter was 4 mm, with a sensitivity of 92%, specificity of 83%, PPV of 86%, NPV of 91%, LR + of 5.43, and LR− of 0.09. The AUCs of the cisterna chyli diameter, age, albumin, sodium, blood urea nitrogen (BUN), BUN/creatinine, c-reactive protein, model for end-stage liver disease score, fractional excretion of sodium, and urinary sodium for evaluating the effect of tolvaptan were 0.911, 0.539, 0.597, 0.551, 0.656, 0.631, 0.547, 0.587, 0.627, and 0.5, respectively. According to a previous study^14^, the cutoff value for BUN was 28.2 mg/dL with a sensitivity of 83%, specificity of 34%, PPV of 57%, NPV of 66%, LR + of 1.27, and LR− of 0.49. With reference to another previous study^27^, the cutoff value for BUN/creatinine was 17.5 with a sensitivity of 38%, specificity of 78%, PPV of 65%, NPV of 54%, LR + of 1.77, and LR− of 0.79. The diagnostic accuracy of other factors predicting the effect of tolvaptan was inferior to that of the cisterna chyli diameter, BUN, and BUN/creatinine. Logistic regression analysis was performed to predict the effect of tolvaptan. In the univariate analyses, the cisterna chyli diameter, cisterna chyli formation, BUN, BUN/creatinine, and furosemide treatment were significant predictive factors (Table 4). In the multivariate analysis, the cisterna chyli diameter and furosemide treatment were significant predictive factors (Table 4).

**Table 3:** Diagnostic accuracy for the effect of tolvaptan.

	AUC	Cutoff	Sensitivity	Specificity	PPV	NPV	LR +	LR−
**Pilot set (n = 100)**								
Cisterna chyli^a^	0.911	4 mm	0.92	0.83	0.86	0.91	5.43	0.09
Age	0.539	51 years	0.22	0.89	0.71	0.50	2.09	0.87
Albumin	0.597	3.1 g/dL	0.34	0.87	0.75	0.54	2.66	0.78
Sodium	0.551	138 mEq/L	0.83	0.32	0.58	0.63	1.22	0.53
BUN	0.656	19.0 mg/dL	0.68	0.62	0.67	0.63	1.77	0.52
BUN/Cr	0.631	18.2	0.47	0.79	0.71	0.57	2.22	0.67
CRP	0.547	3.51 mg/dL	0.83	0.34	0.59	0.64	1.25	0.60
MELD	0.587	13	0.45	0.77	0.69	0.55	1.93	0.71
FENa	0.627	0.68	0.61	0.68	0.70	0.59	1.91	0.57
Urinary sodium	0.561	38 mmol/L	0.45	0.80	0.77	0.54	2.26	0.69
**Validation set (n = 172)**								
Cisterna chyli^a^	0.988	4 mm	0.95	0.97	0.97	0.94	33.1	0.06

*AUC* area under the curve, *PPV* positive predictive value, *NPV* negative predictive value, *LR* + positive likelihood ratio, *LR − *negative likelihood ratio, *BUN* blood urea nitrogen, *BUN/Cr* blood urea nitrogen/creatinine, *CRP* c-reactive protein, *MELD* model for end-stage liver disease, *FENa* fractional excretion of sodium.

^a^The cisterna chyli diameter.

**Table 4:** Predictors of the effect of tolvaptan.

Factors	Univariate analysis			Multivariate analysis		
	Odds ratio	95% CI	p value	Odds ratio	95% CI	p value
Diameter of cisterna chyli ≥ 4 mm	96.2	34.1–271.2	< .001	258.0	29.5–2260.0	0.022
Body weight (> 60 kg)	1.5	0.8–2.6	0.222	164.5	0.3–88,702.8	0.112
Anti-HCV ( +)	0.6	0.3–1.1	0.115	0.0	0.0–2.5	0.097
Male	1.4	0.8–2.4	0.280	555.6	0.6–4862.4	0.068
Age > 65 years	1.0	0.6–1.9	0.907	4.8	0.1–351.0	0.474
Albumin < 2.5 g/dL	0.6	0.3–1.1	0.116	0.1	0.0–4.1	0.191
Na < 130 mEq/L	0.7	0.3–1.8	0.495	0.0	0.0–4.1	0.121
BUN < 28 mg/dL	2.2	1.1–4.7	0.032	8.0	0.0–1494.2	0.434
BUN/Cr < 17.5	2.0	1.0–4.0	0.040	163.1	0.3–80,145.1	0.107
eGFR > 60 mL/min/1.73m^2^	1.1	0.6–2.0	0.824	2.7	0.0–245.8	0.660
CRP < 0.9 mg/dL	1.7	0.9–3.1	0.099	13.2	0.3–592.5	0.183
MELD < 16	1.0	0.5–2.0	0.839	1.4	0.0–61.5	0.853
FENa < 0.35	1.5	0.6–3.8	0.382	2.1	0–456.5	0.853
Urinary sodium > 50 mmol/L	1.3	0.6–2.9	0.489	133.3	0.0–47,694.6	0.103
Furosemide < 40 mg^a^	2.3	1.2–4.3	0.012	27.2	0.5–1612.0	0.113
Spironolactone < 50 mg^b^	0.9	0.5–1.6	0.620	0.2	0.0–7.5	0.356
Esophagogastric varices	0.7	0.4–1.3	0.275	0.0	0.0–3.2	0.106
History of HCC treatment	0.6	0.2–1.8	0.354	0.0	0.0–15.8	0.229

*CI* confidence interval, *HCV* hepatitis C virus, *BUN* blood urea nitrogen, *BUN/Cr* blood urea nitrogen/creatinine, *CRP* c-reactive protein, *MELD* model for end-stage liver disease, *FENa* fractional excretion of sodium, *HCC* hepatocellular carcinoma.

^a^Dose of furosemide during tolvaptan treatment.

^b^Dose of spironolactone during tolvaptan treatment.

### Cumulative liver transplantation-free survival rate

**Figure 2:**
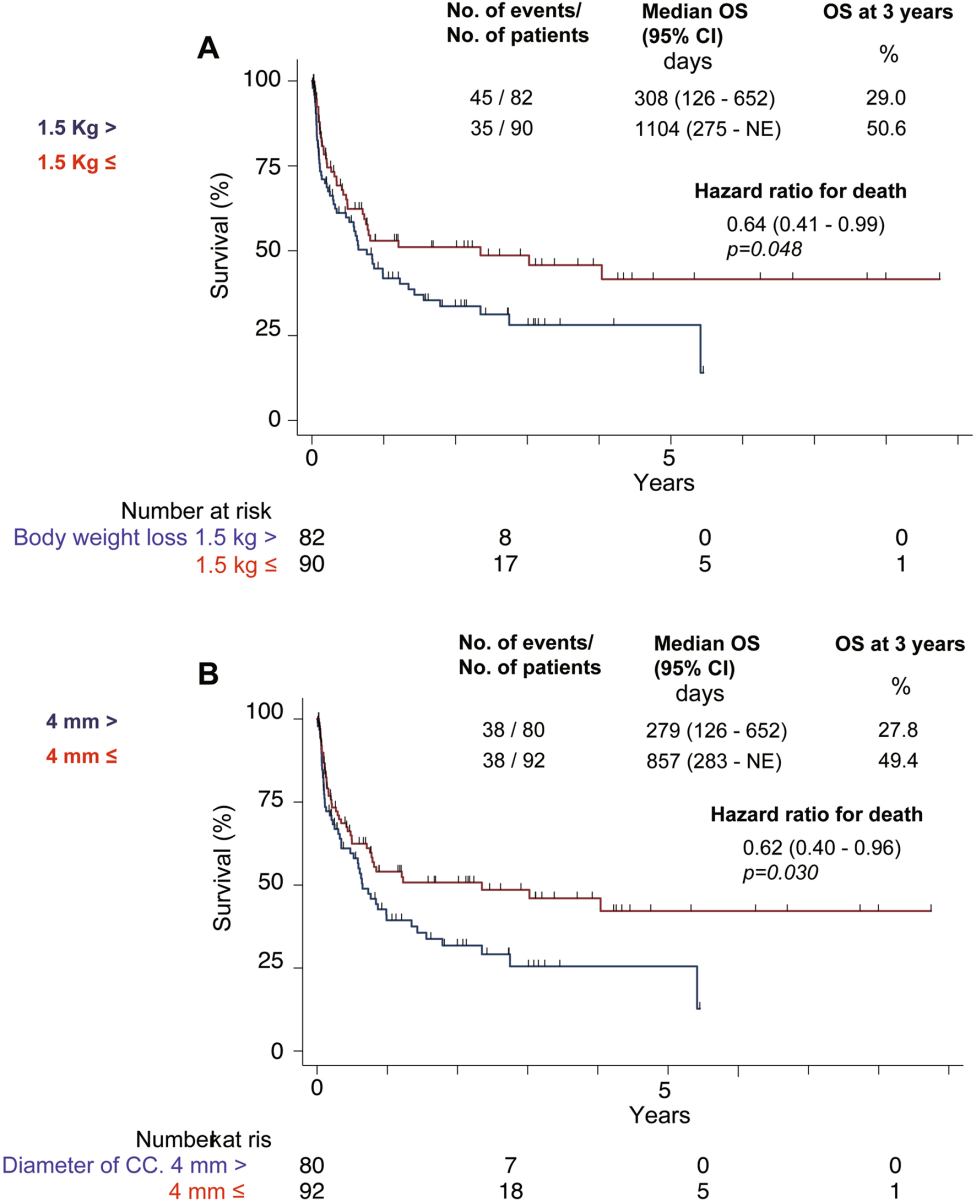
Cumulative liver transplantation-free survival rate. (**A**) Cumulative liver transplantation-free survival rates were significantly higher in the group achieving body weight reduction than in the group with incomplete body weight reduction. (**B**) Cumulative liver transplantation-free survival rates were significantly higher in the group that responded to tolvaptan than in the group not responding to tolvaptan.

Cumulative liver transplantation-free survival rates were significantly higher in patients with a 1.5-kg body weight reduction than in those without (55.4% vs. 44.8% in the first year, 50.6% vs. 29.0% in the third year, and 43.1% vs. 29.0% in the fifth year; p = 0.048; hazard ratio, 0.64 [95% confidence interval (CI) 0.41–0.99]; Fig. 2A). Moreover, cumulative survival rates were significantly higher in patients with cisterna chyli dilatation than in those without (55.6% vs. 45.5% in the first year, 49.4% vs. 27.8% in the third year, and 42.2% vs. 27.8% in the fifth year; p = 0.030; hazard ratio, 0.62 [95% CI 0.40–0.96]; Fig. 2B).

### Adverse effect

Adverse effects are shown in Table 5. All events were infrequent. There was no significant difference in adverse events between the responder and non-responder groups. There was no patient with elevated alanine aminotransferase levels.

**Table 5:** Adverse effect.

Factors	All (n = 172)	Group		p value
		Responders (n = 90)	Non-responders (n = 82)	
Hepatic encephalopathy^a^	5 (2.9%)	4 (4.4%)	1 (1.2%)	0.208
Elevation of ALT^b^	0	0	0	1.000
Hyper natremia > 150 mmol/l	2 (1.2%)	1 (1.1%)	1 (1.2%)	0.947
Hyperkalemia > 5.5 mmol/l	3 (1.7%)	1 (1.2%)	2 (2.2%)	0.606
Renal dysfunction^c^	2 (1.2%)	1 (1.1%)	1 (1.2%)	0.947

^a^More than grade 2 (Lethargy or moderate confusion was occurred).

^b^Creatinine level increased by ≥ 0.3 mg/dL.

^c^Alanine aminotransferase increased more than 1.5-fold.

## Discussion

Certain factors have been reported to predict the efficacy of medical therapy for severe ascites; nonetheless, none has been conclusive. In this study, cisterna chyli dilatation emerged as an optimal marker of treatment response before the initiation of medical treatment.

The relationship between the lymphatic system and hepatic ascites has recently attracted renewed attention. The liver produces vast quantities of lymph, estimated at 25–50% of the lymph, flowing through the thoracic duct^20,28^. In patients with cirrhosis, lymphatic fluid produced in the liver increases up to thirty-folds^29–32^. Lymphatic drainage impairment and interstitial fluid build-up are direct causes of ascites development^20–22^. However, the role of the lymphatic vascular system in the pathogenesis of ascites is yet to be fully elucidated. Therefore, we focused on lymphatic drainage dysfunction before lymph is returned to the systemic circulation^33,34^. Ghelfi et al. reported that patients with cirrhosis and portal hypertension exhibited a significant pressure gradient between the thoracic duct and left subclavian vein and that its correction after thoracic duct stenting might have induced ascites resolution. Thus, this suggests that lymphatic flow deactivation in the thoracic duct potentially aggravates hepatic lymphatic system dysfunction; however, this may not apply to all cases. Although Ghelfi et al. suggested lymphovenous-junction narrowing as a major cause of lymphatic drainage dysfunction, a figure in their study revealed narrowing of the entire thoracic duct. The thoracic duct and cisterna chyli are often reported to increase in diameter with increasing portal pressure^26,35^. However, the cisterna chyli diameter in patients with severe ascites^26^ is noticeably narrower than that in healthy patients^36^. Thus, we hypothesized that the cisterna chyli and thoracic duct, which dilate as portal pressure increases, become narrower as liver disease progresses and fail to tolerate the increased internal pressure, and lymphatic vessel walls lose their ability to extend. Although not proven, similar hypotheses have been postulated in previous studies^26^. In patients with decompensated cirrhosis involving a narrow cisterna chyli (< 4 mm), lymphatic drainage into the systemic circulation is presumably unsuccessful. Additionally, albumin in plasma and water components potentially leaks into the interstitium, further contributing to hypoalbuminemia in patients with decompensated cirrhosis. Unexpectedly, there was only one case of chylous ascites in a patient with a narrowed cisterna chyli. According to Stewart et al.^37^, in patients with narrowed cisterna chyli, intestinal-derived lymph containing a high amount of triglycerides could not be merged with liver-derived lymph. Although MDCT was used in this study, MRI can be used to delineate the cisterna chyli. However, MRI takes more time than CT. Further, MRI may be inappropriate for patients with severe ascites because of signal attenuation. On comparing survival rate with cisterna chyli diameter, the prognosis worsened in patients with a narrower cisterna chyli diameter. This finding is consistent with that in previous reports^26^. The shape of the survival curve approximated that of the curve comparing patients with and without body weight improvement, thus suggesting that in patients with severe ascites, the cisterna chyli diameter is an effective prognostic predictor of ascites. Although various markers have been reported as predictive markers for the medical treatment of severe ascites, none have been definitive. When patients are divided into two groups according to the cisterna chyli diameter (i.e., ≥ or < 4 mm), a significant difference in BUN and BUN/Cr, among other factors, is observed. However, in multivariate analysis, only the cisterna chyli diameter and furosemide treatment were significant predictive factors for the effect on severe ascites treatment. Goto et al. reported a case of effective treatment with tolvaptan after furosemide reduction. They hypothesized that furosemide potentially decreases urine osmolality, resulting in no response to tolvaptan^38^. Hence, in patients not responding to tolvaptan, we hereby propose the initial reduction of furosemide dosage. Moreover, in patients with a narrow cisterna chyli diameter (< 4 mm), we recommend a transition to transjugular intrahepatic portosystemic shunt (TIPS), liver transplantation, and other therapies instead of taking medication indiscriminately.

This study has several limitations. First, there was no validation cohort in other institutes. In the future, a multicenter validation trial should be conducted. The interactions between tolvaptan treatment response and lymphatic function and various other factors that affect ascites formation should also be analyzed with a large number of patients. Second, the pathological mechanism underlying cisterna chyli diameter narrowing in decompensated cirrhosis was not clarified. The irreversibility of this narrowing and its ability to cause lymphatic drainage dysfunction remain unclear. Animal experiments are required to elucidate these mechanisms. Third, the ascites developed in patients enrolled in this study is strictly different from refractory ascites. Because of the small stature of the Japanese population, treatment with tolvaptan is often initiated for patients with low-dose diuretics, as reported by Sakaida et al^39^. In the future, a group of patients with refractory ascites only should be analyzed. Finally, while AP2 and urine osmolality have been reported as predictors of tolvaptan’s effectiveness, they could not be examined in this retrospective study^40^. A prospective study is required to analyze these factors.

In conclusion, cisterna chyli dilatation proved to be an effective optimal marker of tolvaptan’s effectiveness in patients with severe cirrhosis. In cases where the cisterna chyli diameter is less than 4 mm, non-pharmacological treatments (e.g., TIPS) should be actively applied.

## Methods

### Patients and study design

This study followed a retrospective design and was approved by the relevant institutional ethics committee (Ehime University of Medical Science). The study was conducted in accordance with the principles of the Declaration of Helsinki. All the subjects provided written informed consent. Two hundred and eighteen patients with decompensated liver cirrhosis and fluid retention (pleural effusion, ascites, or lower-limb edema) were included in this retrospective study at our hospital between December 2012 and August 2021. The inclusion criteria were as follows: (1) age between 20 and 90 years; (2) non-consideration of sex during the selection process; (3) diagnosis of liver cirrhosis by ultrasound, CT, or liver biopsy; (4) presence of ascites unresponsive to standard therapy, including diuretic therapy comprising furosemide, spironolactone, or both, in addition to the appropriate salt (< 5–7 g/day) restrictions; and (5) patients in whom pre-breakfast body weight was stable (within the range of ± 1 kg). The exclusion criteria, regarding which reference was made to a previous phase 3 trial^41^, were as follows: (1) hepatic encephalopathy stage^42,43^ 2 or higher; (2) vascular invasive hepatocellular carcinoma; (3) esophagogastric varices requiring treatment; (4) a history of cerebrovascular disorder; (5) type 1 hepatorenal syndrome; (6) body mass index > 35; (7) systolic blood pressure < 90 mmHg; (8) hemoglobin < 8.0 g/dL; (9) serum sodium < 120 mEq/L or > 147 mEq/L; (10) serum potassium > 5.5 mEq/L; (11) inability to take oral medication; (12) pregnancy; (13) inability to undergo contrast-enhanced CT; (14) inability to have body weight measured one week after tolvaptan initiation; and 15) eGFR < 30 mL/min/1.73m^2^. All authors had access to the study data and reviewed and approved the final manuscript.

### Treatment protocol

Patients were initially administered salt-restricted diet therapy (5–7 g/day) and conventional diuretics (furosemide, spironolactone, or both) for at least seven days. If body weight remained constant after the aforementioned treatments, tolvaptan (Samsca®, Otsuka Pharmaceutical Co., Ltd., Tokyo, Japan) was orally administered at a dosage of 7.5 mg once a day for at least one week. Water intake was not restricted during the tolvaptan administration, and no albumin preparation was infused. Ascites and pleural effusion were not resolved by paracentesis during the first seven days of tolvaptan treatment. According to previous studies^14,39,44^, patients with a ≥ 1.5-kg decrease in body weight from baseline were defined as responders.

### Laboratory test

Body weight and 24-h urine volume were measured and recorded daily from baseline to one week after treatment. Body weight was measured on wakening. The hematological test items shown in Table 1 were measured at tolvaptan initiation. Additionally, 24-h urine volume, urinary sodium, and creatinine secretion were measured at tolvaptan initiation.

### Imaging study

**Figure 3:**
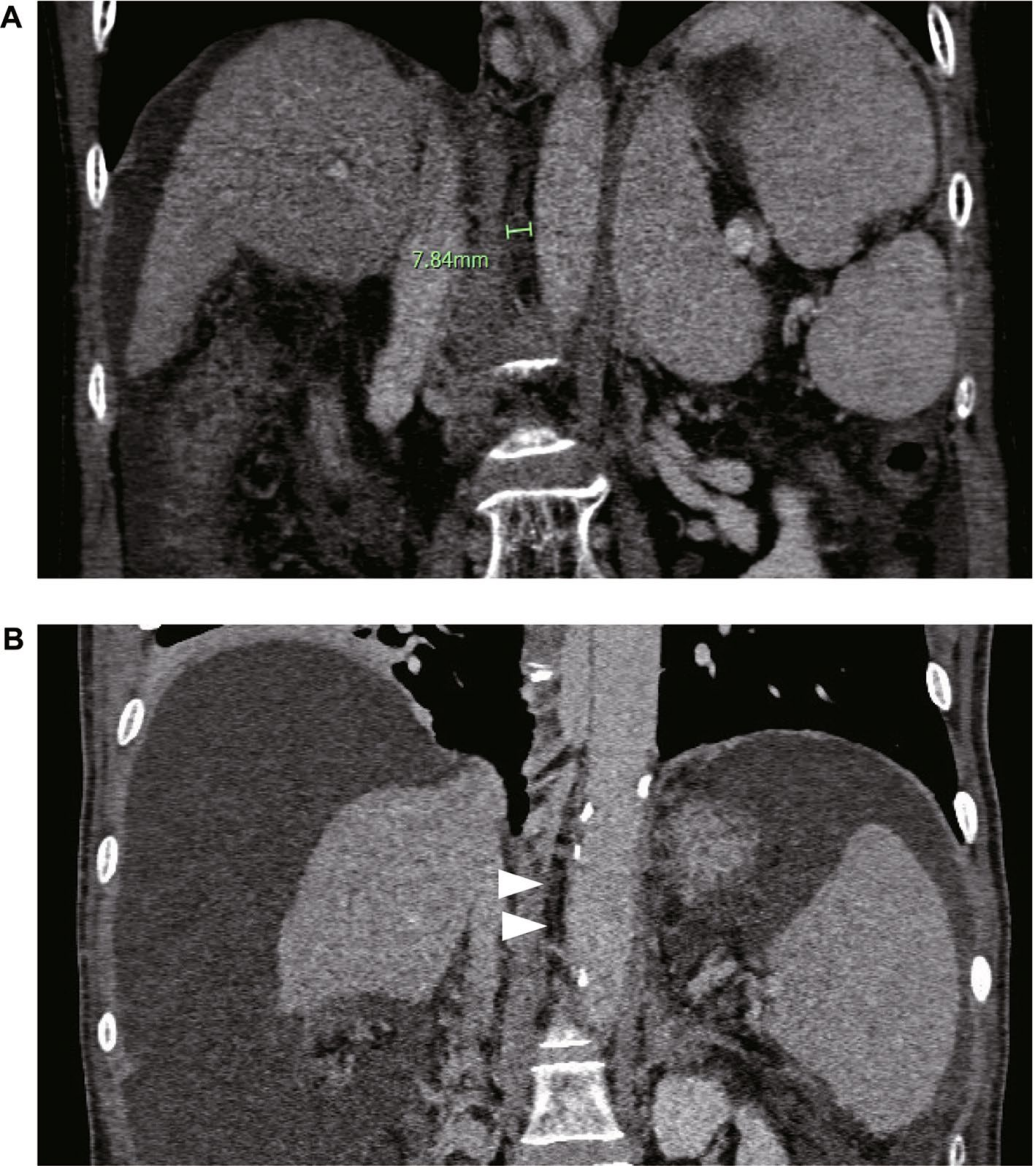
Cisterna chyli patterns. (**A**) Dilatated cisterna chyli (at measure point). (**B**) Narrowing cisterna chyli.

Contrast-enhanced CT scans were acquired in the portal venous phase with patients in a supine position and arms above the head using a 128-slice spiral CT scanner. The patients fasted for at least 3 h before the contrast-enhanced CT examination. The CT images were taken with intermittent breath-holding after maximum inspiration. As in previous studies, the cisterna chyli was assessed^45^. The cisterna chyli was identified in the retrocrural space, right of the aorta, as an oblong structure that is isodense to water, typically extending cranially into the lower-caliber thoracic duct. Between Th12 and L2, the maximum axial diameter of the cisterna chyli was measured independently by two hepatologists, perpendicular to the long axis within four weeks before initiating tolvaptan treatment. Image analysis was performed on the basis of axial slices and additional coronal and sagittal reformation. Measurement of the largest cisterna chyli diameter (outer circumference to outer circumference) was manually performed by both readers (Fig. 3).

### Statistical analysis

After confirmation of data normality using the Shapiro–Wilk test, quantitative data were presented as the median (interquartile range). To compare clinical characteristics and effects between the two groups, the Mann–Whitney *U*-test was applied. Percentages were compared using the *χ*^2^ or Fisher’s exact test. Univariate and multivariate logistic regression analyses were performed to investigate factors predicting the achievement of a 1.5-kg body weight reduction from baseline. Sensitivity, specificity, PPV (positive predictive value), NPV (negative predictive value), LR + (positive likelihood ratio), and LR− (negative likelihood ratio) scores were calculated to evaluate tolvaptan’s efficacy. The AUCs were determined by receiver-operating characteristic curve analysis. To assess survival and recurrence curves, the Kaplan–Meier method was used. All statistical analyses were performed using STATA (version 15; StataCorp, College Station, TX, USA). Statistical significance was set at p < 0.05.

## Data Availability

The data sets generated and/or analyzed in this study are not publicly available because consent for publication has not been obtained from all patients, but are available from the corresponding author upon reasonable request.
